# Cervicothoracic Lordosis Can Influence Outcome After Posterior Cervical Spine Surgery

**DOI:** 10.2174/1874325001812010091

**Published:** 2018-03-16

**Authors:** Albert Vincent Berthier Brasil, Pablo Ramon Fruett da Costa, Antonio Delacy Martini Vial, Gabriel da Costa Barcellos, Eduardo Balverdu Zauk, Paulo Valdeci Worm, Marcelo Paglioli Ferreira, Nelson Pires Ferreira

**Affiliations:** Department of Neurosurgery, Hospital São José – Santa Casa de Misericórdia de Porto Alegre, Porto Alegre, Rio Grande do Sul,

**Keywords:** Cervicothoracic Lordosis, C2-C7 SVA, HRQOL, Posterior Approach, Cervical sagittal balance, Quality of life

## Abstract

**Background:**

Previous studies on the correlation between cervical sagittal balance with improvement in quality of life showed significant results only for parameters of the anterior translation of the cervical spine (such as C2-C7 SVA).

**Objective:**

We test whether a new parameter, *cervicothoracic lordosis*, can predict clinical success in this type of surgery.

**Methods:**

The focused group involved patients who underwent surgical treatment of cervical degenerative disk disease by the posterior approach, due to myelopathy, radiculopathy or a combination of both. Neurologic deficit was measured before and after surgery with the Nurick Scale, postoperative quality of life, physical and mental components of SF-36 and NDI. Cervicothoracic lordosis and various sagittal balance parameters were also measured. Cervicothoracic lordosis was defined as the angle between: a) the line between the centroid of C2 and the centroid of C7; b) the line between the centroid of C7 and the centroid of T6. Correlations between postoperative quality of life and sagittal parameters were calculated.

**Results:**

Twenty-nine patients between 27 and 78 years old were evaluated. Surgery types were simple decompression (laminectomy or laminoforaminotomy) (3 patients), laminoplasty (4 patients) and laminectomy with fusion in 22 patients. Significant correlations were found for C2-C7 SVA and cervicothoracic lordosis. C2-C7 SVA correlated negatively with MCS (r=-0.445, p=0.026) and PCS (r=-0.405, p=0.045). Cervicothoracic lordosis correlated positively with MCS (r=0.554, p= 0.004) and PCS (r=0.462, p=0.020) and negatively with NDI (r=-0.416, p=0.031).

**Conclusion:**

The parameter *cervicothoracic lordosis* correlates with improvement of quality life after surgery for cervical degenerative disk disease by the posterior approach.

## 1 INTRODUCTION

The neurological improvement which is usually observed after cervical spinal cord decompression in cervical spondylotic myelopathy (CSM), may be accompanied by a lack of correspondent improvement in the quality of life [1], mostly due to continuing pain and its associated dysfunctions. While pain measurement scales are not usually good tools to capture and quantify these phenomena [2], the evaluation with some Health-Related Quality of Life (HRQOL) tests such as SF-36 and Neck Disability Index (NDI) demonstrated very clearly the dissociation between a consistent neurological improvement and a relatively unpredictable outcome in terms of dysfunction (NDI) or physical and mental quality of life - Mental Component Summary (MCS) and Physical Component Summary (PCS) of SF-36 [1, 3]. The tremendous progress recently achieved in the understanding of spinopelvic balance –and the associated muscle energy expenditure to maintain posture– was able to improve the surgical planning process in this area and to offer correspondingly improved clinical results [4]. This progress led to a renewed interest on the study of cervical sagittal balance, aiming to offer the same benefits for cervical spine patients. Unfortunately, the advancement in the knowledge about the cervical balance lags behind its thoracolumbar counterpart in a persistently slow pace [2, 3].

Cervical degenerative disc disease (DDD) can be operatively treated by the anterior and the posterior routes. The intuitive, simple and biomechanically plausible concept that cervical lordosis represents a fundamental surgical goal in posterior cervical surgery seems to be the first casualty in this recent surge of interest about cervical spinal balance. Some clinical series demonstrated that the intrinsic cervical lordosis may bear no correspondence with HRQOL after surgery, especially when performed by posterior approach [1, 2, 5-7]. Even more surprising was the report that cervical kyphosis was present and unnoticed in one third of asymptomatic persons [4].

We hypothesized that such a biologically plausible and intuitive concept as the clinical importance of cervical lordosis should not be simply abandoned. Our idea comes from the lumbar area, where lumbar lordosis cannot be considered as an independent surgical goal. The importance of lumbar lordosis can only be considered if one takes into consideration its mechanical and muscular relationships with the supporting pelvic structures [8]. The cervical spine is mainly supported by the upper half of the thoracic spine and by muscles that attach to the latter. One can then hypothesize that the reciprocal relationships between these two segments of the spine might be the determinant of the effectiveness of muscle efforts to maintain posture.

We retrospectively evaluated the data of patients operated in our service in order to verify if a new, simple and intuitive cervical sagittal parameter, which was called cervicothoracic lordosis (CTL) could correlate with the quality of life after the surgical treatment of cervical DDD performed by the posterior approach and help define the surgical strategy.

## 2 MATERIALS AND METHODS

The study was approved by the Ethics Committee of the Santa Casa de Misericordia Hospital Complex and the patients signed an Informed Consent Term (Termo de Consentimento Livre e Esclarecido) authorizing the procedure and the inclusion in the study protocol. Patients from both sexes, without any restriction of age, who were surgically treated in our service between March/2012 and July/2015 of cervical degenerative disk disease by the posterior approach were included. The reasons for the surgeries were myelopathy, radiculopathy or a combination of both problems. As primary outcome, this study has evaluated the effect of cervical sagittal balance on the quality of life of the patients. Secondary outcome measures were the correlation between the new parameter with other sagittal balance measures.

The analysis of postoperative cervical sagittal balance was performed with standing digital x-Rays of the whole spine, including the upper part of the femoral bones inferiorly up to the skull base superiorly. Measurements of angles and distances were performed with the software Surgimap V 2.2.9.6 by a trained physician who was not a part of surgical team and blindly revised by a spine surgeon expert. The following parameters were analyzed: C2-C7 SVA (sagittal vertical axis), Cobb C2-C7, and the new parameter proposed here, Cervicothoracic Lordosis (CTL). Cervicothoracic lordosis was defined as the angle between two lines: a) the line between the centroid of C2 and the centroid of C7; b) the line between the centroid of C7 and the centroid of T6 Fig. (**1**). This angle was considered positive when it was positioned in lordosis and negative when in kyphosis. Millimeters and degrees were rounded to the unit.

Neurologic deficit was measured with the Nurick Scale [9]. Health related quality of life was measured with the use of the Short-Form-36 (SF-36) [10], and the Neck Disability Index (NDI) [11]. Questionnaires were applied by a trained research assistant and answered by the patients. They were answered in the preoperative day and in the last clinical evaluation, on the day when X-Rays were performed. The statistical analysis data from the SF-36 was summarized by the Physical Component (PCS) and the Mental Component (MCS) Summary Scores [12].

Data were presented in absolute (n) and relative (%) frequencies. Numeric data were expressed as mean and standard deviation. Normality was tested with the Komogorov-Smirnov test. We tested the correlation between the parametric variables with the Pearson’s correlation test and nonparametric variables with the Spearman correlation test. We considered significant the values of “p” lower than 0.05. Data analysis was performed with the Statistical Packages for Social Sciences v15.0 (SPSS, Chicago, IL).

## 3 RESULTS

A total of 29 patients was evaluated and the time for postoperative evaluation with X-Rays and HRQOL questionnaires was 12±5 months. The descriptive analysis of the patient sample is presented in Table (**1**).

There was a small but significant improvement in Nurick grade after surgery from 1.43±1.34 to 1.00±1.22 (p=0.05). NDI, PCS and MCS were measured preoperatively (NDI: mean 3.19 + 1.25; PCS - median 23.84 min=10, max=58; MCS: mean 46.9±14.609) and in the last evaluation (NDI: mean 2.81±1.10, MCS: mean 46.90±14.60, and PCS [nonparametric distribution] varied between 10 and 58 with a median value of 23.84). The postoperative improvement in PCS and MCS was statistically significant but the improvement in NDI was not. Improvement in PCS was significant [13, 14] (>5 points) in twelve patients (57%) and minimally clinically significant (>2.6 and <5) in one (5%). No improvement (<2.6 points) was detected in eight patients (38%). Significant improvement in MCS was observed in eleven patients (52%), minimally clinically significant improvement was detected in one patient (5%) and no improvement in 9 (43%). In 6 patients (25%) NDI improved significantly (more than 8.4 points), while in seven (29%), the improvement was minimally clinically significant (>3 and < 8.4). Eleven patients (46%) had no improvement in NDI [15].

The values of C2-C7 SVA, Cobb C2-C7 and CTL are presented in Table (**1**). Correlations between radiographic parameters and HRQOL measurements are shown in Table (**2**). Significant correlations were found exclusively for C2-C7 SVA and CTL. No correlation between Cobb C2-C7 and HRQOL measurements was detected.

## 4 DISCUSSION

Many parameters have been used to describe and study the cervical sagittal balance [3, 4, 16]. Attempts to correlate these parameters with clinical success after surgery were published by a few authors but most of the parameters showed no correlation with clinical data. Parameters that measure the anterior translation of the cervical spine like C2-C7 SVA stand almost alone as the only indicators for good outcome after surgery [1, 2]. The present findings demonstrate that the new parameter, CTL, correlates positively with HRQOL after surgery for cervical DDD by the posterior approach. Findings were significant for the Physical and the Mental Component Scores of the SF-36 as well as for NDI. In our series, C2-C7 SVA showed the same type of correlation for PCS and MCS of SF-36 but not for NDI. All other parameters examined, including cervical lordosis, showed no correlation with quality of life Table (**2**). Our findings are in accordance with previous authors who found a similar correlation for C2-C7 SVA but not for all other parameters [1, 2]. We did not find any mention to cervicothoracic lordosis in previous literature.

The concept of cervicothoracic lordosis proposed here was designed after a careful analysis of the previous data from the literature. Samundrala [17] and Deviren [18] described the correction of cervicothoracic deformity in cases that included post laminectomy and degenerative kyphosis. These authors performed PSO at C7 or T1 aiming to improve cervicothoracic lordosis (although they did not explicitly say so). The correction of this angle was accompanied by significant improvement in NDI and in the Physical and Mental components of SF-36. In fact, a careful review of the clinical cases presented the literature devoted to “postoperative cervical kyphosis” shows many patients who presented a decrease in cervicothoracic lordosis and not of intrinsic cervical lordosis [19-21].

Other authors tried to improve the understanding of cervical sagittal balance by including its relationships with neighboring thoracic or cranial structures. An attempt to replicate the model of Pelvic Index and its associated variables for the cervical spine was published by Lee *et al*[16]. This model is based on the assumption that Thoracic Inlet Angle (TIA) is an anatomical constant, in the same fashion that the pelvic index. No study correlating these variables with HRQOL was published so far. The idea that TIA is a constant was threatened by Janusz *et al*. [22] who demonstrated that it can vary with the patient’s position. Le Huec [4] tried to expand the knowledge in this field with the study of many craniocervical angles of normal asymptomatic persons. The analysis of 106 subjects led to the conclusion that the craniospinal angle should be in the range of 83±9 degrees in order to maintain normal energy expenditure in cervical muscles. It is interesting to note that this attempt to study reciprocal relations between the cervical spine and the cranium leads to a conclusion linking the cervical spine to the upper thoracic spine. In the same fashion that cervicothorcic lordosis, craniospinal angle represents the angle between the cervical spine axis and the inferior endplate of C7 which is an indirect representative of the axis of the upper thoracic spine or thoracic kyphosis.

The mechanics of C2-C7 SVA and CTL are simple. To understand the phenomena described here, one has to consider a mechanical system composed of one heavy object to be maintained in position (the head), one passive lever arm (the cervical spine as a whole), one active component (the erector spinae muscles), one supporting point for the lever arm (the superior endplate of T1) and the attachments of the muscles at the cervical spine at one end and at the superior half of the thoracic spine at the other end. As C2-C7 SVA increases, the moment of force applied by the weight of the head on the cervicothoracic junction increases proportionally Fig. (**2**). As cervicothoracic lordosis increases, the force vector applied by the muscles becomes progressively more perpendicular to the passive lever arm (more efficient) Fig. (**3**). In simple terms, C2-C7 SVA seems to measure the “weight of the head on the cervicothoracic junction”, while cervicothoracic lordosis represents how efficiently muscles apply the force necessary to support this weight.

Understanding the mechanics of C2-C7 SVA and cervicothoracic lordosis might be of help while planning surgery for cervical DDD, especially when planning instrumentation associated to cervical laminectomy. Surgeons have always been concerned about preserving or improving cervical lordosis in cervical laminectomies. The addition of instrumentation is widely accepted as a solution to this problem [23, 24]. In order to improve lordosis, some surgeons recommend extending the patient’s neck after laminectomy and before definitive contouring of the rods [25]. This maneuver will generally have a lordosing effect mostly on the mobile upper cervical spine (C1-C2) and very little effect between C3 and C7, which are responsible for a very little percentage of global cervical lordosis [26, 27]. The consequence of this fact is that, although some lordosis is gained, it has very little effect in terms of improving C2-C7 SVA. The issue of choosing the distal level of instrumentation in Cervical Laminectomy with Fusion is rarely discussed. Options include limiting the fusion only to the laminectomized vertebrae or extending it distally for one or more levels. Ending a fixation at C6 or C7 is probably useful to preserve intrinsic cervical lordosis. Unfortunately, it has been proven that preserving the intrinsic cervical lordosis seems to be of minor importance in terms of quality of life [1, 2, 6, 7].

Our findings present evidence in favor of a different strategy. Instead of cervical lordosis, preserving or regaining cervicothoracic lordosis could become a surgical goal. In cases where preoperative cervicothoracic lordosis is preserved, extending the instrumentation down to T2 or T3 might be a better option to prevent postoperative kyphosis than just ending the instrumentation at C6 or C7. In cases where preoperative cervicothoracic lordosis is low or negative, an attempt to improve this angle – by C6 and/or C7 Smith-Petersen osteotomy or C7 or T1 Pedicle Subtraction Osteotomy – could prove to be a good policy. Of course, these osteotomies have to be associated to fusion down to T2 or T3.

Many limitations can be identified in the present study. The first one is the absence of a register of the preoperative angles. Of course these numbers would add light to the presented findings. The second is the small size of the sample. This problem is due to the fact that it was a single institution series. Although statistical analysis was possible with sound results, a larger sample would possibly allow the analysis of the questions raised by our conclusion. For example: what is the “appropriate” or “minimally acceptable” value for CTL in order to achieve a good clinical result? Another limitation -which is common to other publications - is the difficulty to separately quantify the additive effects of neurological gains plus sagittal balance gains or losses in the final HRQOL after posterior surgery for cervical DDD.

The present findings need to be further studied. To the authors, it seems that cervicothoracic lordosis can eventually take the place of cervical lordosis in the traditional list of surgical priorities of posterior cervical laminectomy with fusion. Attention to cervicothoracic lordosis probably represents an opportunity to deal with postoperative deformity in its most incipient phase.

## CONCLUSION

The parameter “cervicothoracic lordosis” correlates with HRQOL after surgery for cervical degenerative disk disease by the posterior approach.

## Figures and Tables

**Fig. (1):**
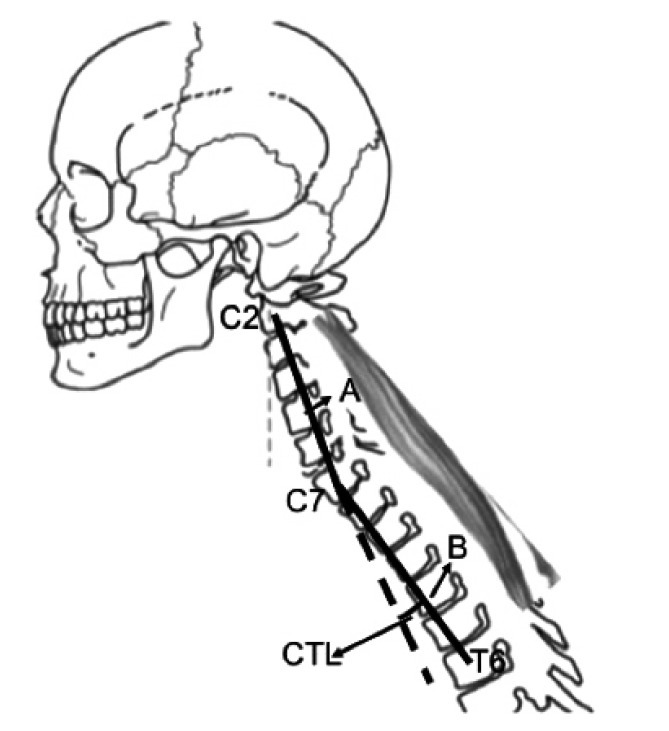
Measure of Cervicothoracic Lordosis. A) the line between the centroid of C2 and the centroid of C7; B) the line between the centroid of C7 and the centroid of T6.

**Fig. (2):**
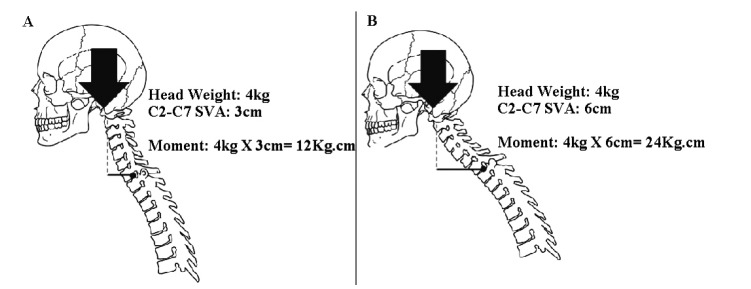
Changes in the moment of force exerted by the head’s weight on the upper extremity of the thoracic spine vary according to C2-C7 SVA. A) Small C2-C7 SVA = small moment. B) large C2-C7 SVA = large moment.

**Fig. (3):**
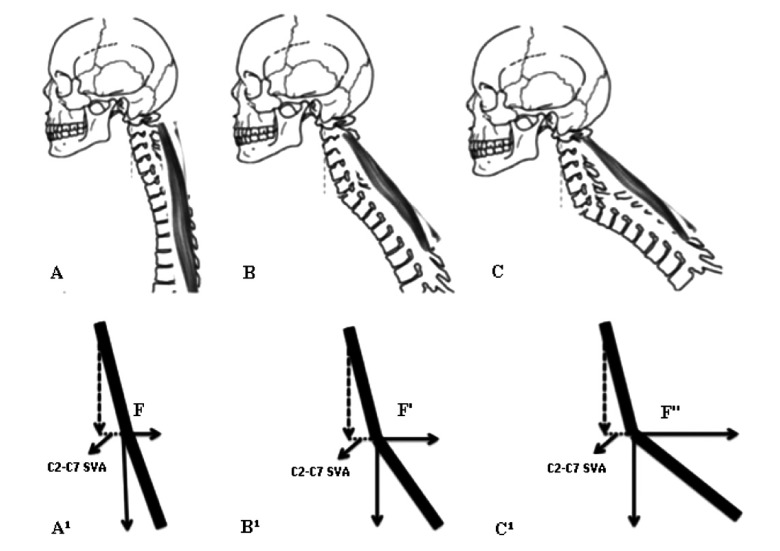
C2-C7 SVA is the same in A^1^ B^1^ and C^1^. A and A^1^: when CTL it is close to zero, the contraction of the spinae erector muscles generates a vector (F) too small in the extension of cervical spine. B and B^1^, C and C^1^: As Cervicothoracic lordosis magnifies, the resultant vector in the extension of the cervical column magnifies proportionally (F' e F “) improving the effectiveness of the muscular contraction and diminishing the muscular energy expense.

**Table 1:** Details of the Patients.

**Gender**				
**Male**	19			
**Female**	10			
**Age**	Range	Mean	SD	
	27-78	54.59	2.249	
**Disease**	Frequency	Percent		
**Myelopathy**	16	55%		
**Radiculopathy**	5	17%		
**Myelopathy + Radiculopathy**	8	28%		
**Types of Surgery**	Frequency	Percent		
**Simple decompression**	3	10.3%		
**Laminoplasty**	4	13.8%		
**Decompression with fusion**	22	75.9%		
**Operated levels**	Levels	Frequency	Percent	
	1	3	10.3%	
	3	7	24.1%	
	4	11	37.9%	
	5	4	13.8%	
	6	3	10.3%	
	9	1	3.4%	
**Post-operative Sagittal Alignment Measures**	n	Minimum	Maximum	Results (M ± SD)
**Cervicothoracic lordosis^£^**	29	-35	20	4 (1 - 6)
**C2-C7 SVA**	29	2	69	29.52 ± 14.60
**Cobb C2-C7**	29	-29	39	4.07 ± 14.26
**Cervicothoracic lordosis^£^**	29	-35	20	4 (1 - 6)

M: mean; SD: standard deviation. ^£^ Result presented in medium and confidence interval of 95%.

**Table 2:** Correlation between HRQOL and Sagittal measures.

	**PCS^£^**	**MCS^£^**	**NDI**	**C2-C7 SVA**	**CTL**	**Cobb C2-C7**
**PCS^£^**	1					
**MCS^£^**	0.66**	1				
**NDI**	-0.82**	-0.67**	1			
**C2-C7 SVA**	-0.40*	-0.44*	0.20	1		
**CTL**	0.46*	0.55**	-0.42*	-0.55**	1	
**Cobb C2-C7**	0.11	0.01	-0.12	-0.53**	0.60**	1

^£^ Post operatory values; * p < 0.05; ** P < 0.01. HRQOL (Health Related Quality of Life), PCS (Physical component summary of SF-36), MCS (Mental component summary of SF-36), NDI (Neck Disability Index), CTL (Cervicothoracic lordosis).

## LIST OF ABBREVIATIONS

C2-C7 SVA: = C2-C7 Sagittal Vertical Axis
Cobb C2-C7: = C2-C7 Cobb Angles
CSM: = Cervical Spondylotic Myelopathy
CTL: = Cervicothoracic Lordosis (CTL)
**HRQOL**: = Health-Related Quality of Life
MCS: = Mental Component Summary of SF-36
NDI: = Neck Disability Index (NDI)
PCS: = Physical Component Summary (PCS) of SF-36
SF-36: = Short-Form-36
TIA: = Thoracic Inlet Angle (TIA)
